# Carbonic Anhydrases as Potential Targets Against Neurovascular Unit Dysfunction in Alzheimer’s Disease and Stroke

**DOI:** 10.3389/fnagi.2021.772278

**Published:** 2021-11-16

**Authors:** Nicole Lemon, Elisa Canepa, Marc A. Ilies, Silvia Fossati

**Affiliations:** ^1^Alzheimer’s Center at Temple (ACT), Lewis Katz School of Medicine, Temple University, Philadelphia, PA, United States; ^2^Department of Pharmaceutical Sciences and Moulder Center for Drug Discovery Research, Temple University School of Pharmacy, Temple University, Philadelphia, PA, United States

**Keywords:** Alzheimer’s disease, stroke, carbonic anhydrase (CA), neurovascular unit (NVU), cerebrovascular pathology, amyloid beta, inflammation, mitochondria

## Abstract

The Neurovascular Unit (NVU) is an important multicellular structure of the central nervous system (CNS), which participates in the regulation of cerebral blood flow (CBF), delivery of oxygen and nutrients, immunological surveillance, clearance, barrier functions, and CNS homeostasis. Stroke and Alzheimer Disease (AD) are two pathologies with extensive NVU dysfunction. The cell types of the NVU change in both structure and function following an ischemic insult and during the development of AD pathology. Stroke and AD share common risk factors such as cardiovascular disease, and also share similarities at a molecular level. In both diseases, disruption of metabolic support, mitochondrial dysfunction, increase in oxidative stress, release of inflammatory signaling molecules, and blood brain barrier disruption result in NVU dysfunction, leading to cell death and neurodegeneration. Improved therapeutic strategies for both AD and stroke are needed. Carbonic anhydrases (CAs) are well-known targets for other diseases and are being recently investigated for their function in the development of cerebrovascular pathology. CAs catalyze the hydration of CO_2_ to produce bicarbonate and a proton. This reaction is important for pH homeostasis, overturn of cerebrospinal fluid, regulation of CBF, and other physiological functions. Humans express 15 CA isoforms with different distribution patterns. Recent studies provide evidence that CA inhibition is protective to NVU cells *in vitro* and *in vivo*, in models of stroke and AD pathology. CA inhibitors are FDA-approved for treatment of glaucoma, high-altitude sickness, and other indications. Most FDA-approved CA inhibitors are pan-CA inhibitors; however, specific CA isoforms are likely to modulate the NVU function. This review will summarize the literature regarding the use of pan-CA and specific CA inhibitors along with genetic manipulation of specific CA isoforms in stroke and AD models, to bring light into the functions of CAs in the NVU. Although pan-CA inhibitors are protective and safe, we hypothesize that targeting specific CA isoforms will increase the efficacy of CA inhibition and reduce side effects. More studies to further determine specific CA isoforms functions and changes in disease states are essential to the development of novel therapies for cerebrovascular pathology, occurring in both stroke and AD.

## Introduction

Neurovascular dysfunction is an important, early and causative event in the pathogenesis of both Alzheimer’s disease (AD) and Stroke (Iadecola, 2010, 2017; Hachinski et al., 2019; Sweeney et al., 2019a; Freitas-Andrade et al., 2020; Zlokovic et al., 2020). Indeed, the Neurovascular Unit (NVU) has recently gained a lot of momentum as a pharmacological target in cerebrovascular pathologies and neurodegeneration (Iadecola, 2017; Sweeney et al., 2019a; Zlokovic et al., 2020). The NVU is a functional multicellular structure composed of blood vessels and different cell types surrounding them within the central nervous system (CNS), and is instrumental in regulating CNS homeostasis (Andreone et al., 2015; Iadecola, 2017; Sweeney et al., 2019b; Freitas-Andrade et al., 2020). Important functions of the NVU include regulation of cerebral blood flow (CBF) and immunological surveillance, amongst others (Andreone et al., 2015; Cortes-Canteli and Iadecola, 2020; Freitas-Andrade et al., 2020). NVU dysfunction is observed in aging, AD and following neurological pathologies such as stroke (Iadecola, 2017; Cortes-Canteli and Iadecola, 2020; Freitas-Andrade et al., 2020; Sarvari et al., 2020) and traumatic brain injury (Xing et al., 2012; Lok et al., 2015), among others.

Stroke and dementia are the two most common neurological disorders. They confer risks for each other and share some, mostly modifiable, risk factors. Having a stroke doubles the chance of developing dementia (Savva and Stephan, 2010; Kuźma et al., 2018). Therefore, preventing stroke through management of hypertension and other risk factors could also decrease the incidence of dementia (Kuźma et al., 2018; Hachinski et al., 2019).

AD is the most common form of dementia and has been historically characterized by extracellular amyloid beta (Aβ) plaques and intracellular hyperphosphorylated tau tangles in specific brain regions (Day et al., 2015; Castillo-Carranza et al., 2017; Cortes-Canteli and Iadecola, 2020; Ojo et al., 2021). Interestingly, Aβ and tau intermediate aggregation species, such as oligomers, have been shown to have toxic effects on multiple cell types of the NVU (Fossati et al., 2010, 2012b; Parodi-Rullán et al., 2019; Canepa and Fossati, 2020). This toxicity, in association with the contribution of impaired clearance of undesired material from the brain, may lead to neurodegeneration and cognitive decline (Fossati et al., 2012b; Boland et al., 2018; Da Mesquita et al., 2018; Nortley et al., 2019; Provensi et al., 2019; Braun and Iliff, 2020; Carare et al., 2020; Cortes-Canteli and Iadecola, 2020; Nedergaard and Goldman, 2020; Parodi-Rullán et al., 2020; Quintana et al., 2021). Importantly, up to 90% of AD patients also present with cerebral amyloid angiopathy (CAA), defined as Aβ deposition around the brain vasculature and/or within the vessel walls (Iadecola, 2017; Sweeney et al., 2019a; Zlokovic et al., 2020). CAA is also common in the non-demented elderly population and constitutes an important contributor to NVU dysfunction in both normal aging and AD (Provensi et al., 2019; Cortes-Canteli and Iadecola, 2020; Ojo et al., 2021).

Stroke is classically defined as a neurological damage attributed to an acute focal injury of the CNS by a vascular cause, including cerebral infarction, intracerebral hemorrhage (ICH), and subarachnoid hemorrhage (SAH), and is a major cause of disability and death worldwide (Sacco et al., 2013). The most common type of stroke is Ischemic Stroke (IS) which occurs when atherosclerotic plaques and fatty deposits cause vascular occlusions, interrupting blood flow in the brain. The blood vessel most commonly occluded is the middle cerebral artery (Kuriakose and Xiao, 2020). When IS occurs, it promptly causes multiple detrimental cerebral injuries due to both the lack of oxygen and glucose, as well as the associated pro-inflammatory signaling (Faraco et al., 2007; Freitas-Andrade et al., 2020; Kuriakose and Xiao, 2020; Sarvari et al., 2020). Following this event, there is a reperfusion injury phase, which occurs when oxygen and CBF are restored (Faraco et al., 2007; Freitas-Andrade et al., 2020; Sarvari et al., 2020). Another type of stroke is hemorrhagic stroke (HS), which occurs when a blood vessel, providing blood to the brain, ruptures (Corraini et al., 2017; Sarvari et al., 2020). HS is characterized by greater lesion volume, higher intracranial pressure and induce more severe brain injury than IS. Importantly, IS and HS affect different brain regions (Corraini et al., 2017).

Shared risk factors between AD and stroke are reduced CBF, cardiovascular diseases and age (Sarvari et al., 2020; Ojo et al., 2021). Cardiovascular risk factors like obesity, diabetes, hypertension and atherosclerosis have been observed to exacerbate cerebrovascular pathology as well as neurodegeneration, including AD (Girouard and Iadecola, 2006; de Bruijn and Ikram, 2014; Cortes-Canteli and Iadecola, 2020; Sarvari et al., 2020). The common underlying mechanisms involved in both AD and stroke include neuroinflammation, mitochondrial dysfunction, cell death, and blood brain barrier (BBB) dysregulation, indicating that the NVU is a target for both diseases (Fossati et al., 2012b; Alluri et al., 2014; Sekerdag et al., 2018; Eldahshan et al., 2019; Provensi et al., 2019; Parodi-Rullán et al., 2020). A vast amount of research has been invested into discovering new treatments for AD and stroke. In AD, this has recently led to the controversial FDA-approval of aducanumab (Ferrero et al., 2016; Sevigny et al., 2017; Knopman et al., 2021). However, more research needs to be done to develop successful disease-modifying therapies for both disorders. The scientific community is particularly encouraging the study of repurposed drugs, approved by the FDA for other disorders, which could be beneficial for AD and stroke, while allowing more rapid translation to clinical trials.

This review will introduce the idea of potentially repurposing carbonic anhydrase inhibitors (CAIs), many of which are already FDA-approved for other indications, for prevention of cerebrovascular and neurovascular pathology in AD and stroke and highlight the impact of carbonic anhydrase (CA) modulation in these two dominant neurological disorders.

CAs are a family of zinc metalloenzymes which catalyze the reversible hydration of carbon dioxide to produce bicarbonate and a proton (CO_2_ + H_2_O ↔ HCO_3_^–^ + H^+^) (Supuran, 2011; Mishra et al., 2020). This chemical reaction is essential for many physiological processes, such as pH and ion homeostasis, carbon dioxide transport, electrolyte secretion, gluconeogenesis, lipogenesis, and ureagenesis, water and sodium reabsorption in the kidney, bone reabsorption and calcification, cerebrospinal fluid formation and turnover, amongst other processes (Provensi et al., 2019; Zamanova et al., 2019; Mishra et al., 2020). CAs have been studied as a well-known pharmacological target for many peripheral and CNS disorders (Bradwell et al., 1992; Ilies et al., 2004; De Simone and Supuran, 2007; Supuran, 2008, 2018; Akocak and Ilies, 2014; Provensi et al., 2019; Zamanova et al., 2019; Mishra et al., 2020). Interestingly, many recent studies revealed a common goal to further elucidate CAs involvement in both AD and stroke.

Humans have 15 CA isoforms, all with different expression patterns at a tissue and cellular level (Supuran, 2011; Provensi et al., 2019; Zamanova et al., 2019; Mishra et al., 2020). Many of these isoforms are expressed within the NVU and it has been hypothesized that each is involved in different functions (Draghici et al., 2014; Rasmussen and Boedtkjer, 2018; Provensi et al., 2019; Zamanova et al., 2019). It is known that some isoforms are extracellular, anchored to the plasma membrane (CA-IV, CA-IX, and CA-XII, CA-XIV), while others are cytosolic (CA-I, CA-II, CA-III, CA-VII, CA-XIII), two are found in the mitochondria (CA-VA and CA-VB) (Boriack-Sjodin et al., 1995; Nishimori et al., 2005), some are acatalytic isoforms (CA-VIII, CA-X, and CA-XI) (Aspatwar et al., 2014), and one isoform is secreted in saliva (CA-VI) (Nishimori et al., 2007; Alterio et al., 2012; Patrikainen et al., 2014; Provensi et al., 2019; Zamanova et al., 2019; Mishra et al., 2020). CA function has been linked to AD pathology as well as stroke, but the specific isoforms and the pathological mechanisms involved are not fully understood (Wang et al., 2009; Draghici et al., 2014; Fossati et al., 2016; Pollard et al., 2016; Solesio et al., 2018; Mishra et al., 2020). The determination of each CA isoforms’ role in health and disease should be a priority for the development of novel and effective therapies in cerebrovascular pathology (Provensi et al., 2019; Mishra et al., 2020).

CAIs were developed first as diuretics and have become valuable in treating glaucoma, cerebral edema, epilepsy, as well as high altitude sickness (Mincione et al., 2007; Supuran, 2008; Ritchie et al., 2012; Akocak and Ilies, 2014; Zamanova et al., 2019; Mishra et al., 2020). The FDA-approved CAIs methazolamide (MTZ) and acetazolamide (ATZ) are the most studied pan-CAIs. Their activity in the NVU will be one of the focuses of this review. Other FDA-approved CAIs such as topiramate, which has some selectivity for the mitochondrial CA-VA and CA-VB isoforms, along with compounds with selectivity for CA-IX and CA-XII will also be discussed (Scozzafava et al., 2000; Supuran, 2012; Andring et al., 2020; McDonald et al., 2020). MTZ and ATZ, along with topiramate, have been observed to have protective properties on cerebrovascular pathology, as well as on mitochondria function, an important target for NVU integrity (Wang et al., 2009; Price et al., 2012; Fossati et al., 2016; Solesio et al., 2018; Salameh et al., 2019). A substantial amount of literature on the FDA-approved pan-CAIs indicates that they are safe and can pass the BBB. However, the development of new compounds targeting specific isoforms may improve the efficacy and reduce side effects of the already existing pan-CAIs (Provensi et al., 2019; Mishra et al., 2020).

This review will first illustrate the basic structure and functions of the cells composing the NVU, specifically describing how they become dysfunctional in stroke and AD. We will then discuss the properties of multiple CA isoforms and highlight the available evidence showing how CA inhibition may be protective toward multiple dysregulated mechanisms in NVU-composing cells, pointing to CAs as potential targets for both stroke and AD therapy (Provensi et al., 2019; Mishra et al., 2020).

## The Neurovascular Unit: Function and Dysfunction in Stroke and Alzheimer’s Disease

### The Neurovascular Unit

**FIGURE 1:**
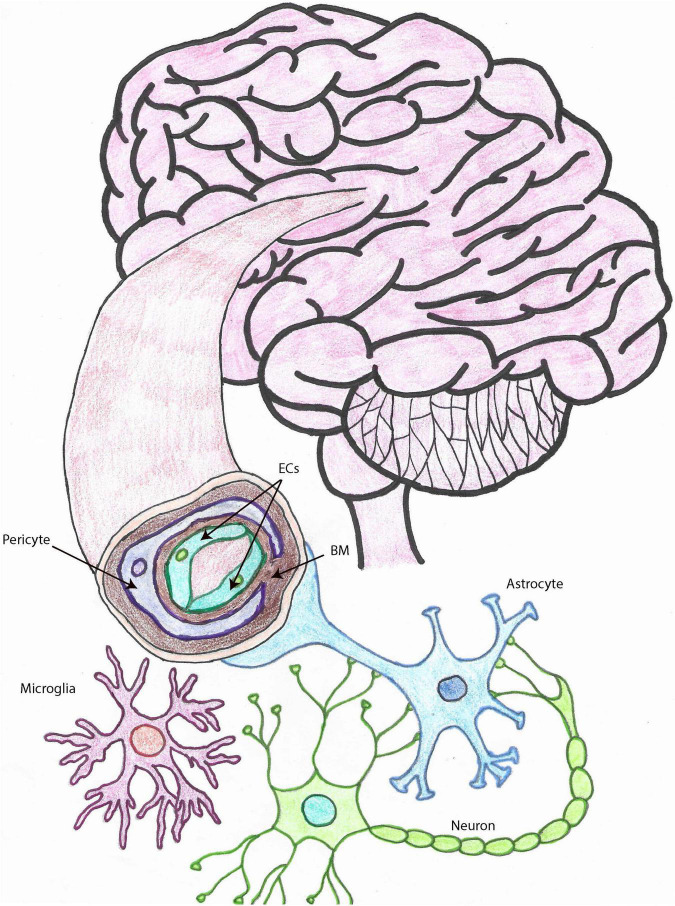
The neurovascular unit. Drawing depicting the cell types that make up the NVU within brain capillaries. Brain capillaries are surrounded by pericytes, as shown in this figure, while arteries and arterioles are surrounded by SMCs. Other important cells associated with blood vessels and important for BBB and neurovascular functions, also represented in this drawing, are astrocytes and microglia. ECs, endothelial cells; SMCs, smooth muscle cells; BBB, blood brain barrier; NVU, neurovascular unit; MCs, microglia cells, BM, basement membrane.

The cell types that constitute the NVU (depicted in Figure 1) and collaborate to perform its functions are endothelial cells (ECs), pericytes, smooth muscle cells (SMCs), astrocytes, and microglia (Iadecola, 2017; Freitas-Andrade et al., 2020), which are functionally or physically connected to neurons (Andreone et al., 2015; Cortes-Canteli and Iadecola, 2020; Freitas-Andrade et al., 2020). The NVU is the morpho-functional unit including the BBB, which is important for the transport of nutrients and oxygen from the systemic circulation to the brain, for the clearance of toxic waste from the CNS, for the connection between blood flow and neuronal function, as well as for forming a physical barrier to prevent the entrance of pathogens and other harmful entities into the CNS (Sarvari et al., 2020).

### Endothelial Cells

ECs are essential components of the blood vessel wall. Cerebrovascular ECs form tight and adherent junctions with each other to limit the entry of molecules and cells from the peripheral circulation into the CNS (Reese and Karnovsky, 1967). Transporters expressed on the plasma membrane of ECs specifically regulate what enters and exits the CNS. For example, glucose enters the brain exclusively via transporters, despite the brain being a highly metabolic organ responsible for up to 25% of total glucose consumption in the body (Tang et al., 2017; Parodi-Rullán et al., 2019). ECs also regulate blood flow by releasing vasodilators and vasoconstrictors, such as nitric oxide (NO) and endothelin-1, respectively (Morikawa et al., 1994; Biernaskie et al., 2001; Freitas-Andrade et al., 2020). Due to the fact that mitochondria are very abundant in cerebrovascular ECs (Oldendorf et al., 1977; Sarvari et al., 2020), these cells are particularly sensitive to oxygen deprivation (Pun et al., 2009; Freitas-Andrade et al., 2020). Hence, pathological conditions which cause oxygen-glucose deprivation (OGD) and prompt excessive reactive oxygen species (ROS) production trigger cerebral endothelial dysfunction, cell death and BBB breakdown (Schreibelt et al., 2007; Pun et al., 2009; Lochhead et al., 2010; Ghiso et al., 2014; Fossati et al., 2016; Parodi-Rullán et al., 2019; Freitas-Andrade et al., 2020), pointing to the mitochondria as critical targets for EC function and BBB integrity. Efficient communication and exchange of materials between ECs and other cell types of the NVU is essential for CNS homeostasis (Andreone et al., 2015; Freitas-Andrade et al., 2020; Sarvari et al., 2020).

Cerebrovascular dysfunction has been observed in both AD and IS (Fossati et al., 2010, 2012a,b; Guo et al., 2010; Lochhead et al., 2010; Jiao et al., 2011; Shin et al., 2016; Freitas-Andrade et al., 2020; Parodi-Rullán et al., 2020; Quintana et al., 2021). Aging, as well as cardiovascular risk factors, such as hypertension and diabetes, contribute to cerebrovascular pathology (Huang et al., 1995; Girouard and Iadecola, 2006; Price et al., 2012; Cortes-Canteli and Iadecola, 2020; Ojo et al., 2021). The reduction of tight junction proteins such as zona occludin-1 (ZO-1) and occludin is observed in models of stroke and AD (Marco and Skaper, 2006; Jiao et al., 2011; Engelhardt et al., 2014; Freitas-Andrade et al., 2020; Parodi-Rullán et al., 2020). An increase in adhesion molecules expression, such as intercellular adhesion molecule-1 (ICAM-1) and vascular cell adhesion molecule-1 (VCAM-1), is observed in stroke, and recently, has been also associated with AD (Cruz Hernández et al., 2019; Sarvari et al., 2020). The reduction of tight junction proteins and the increase in adhesion molecules triggers the recruitment of peripheral immune cells into the brain and may be due to endothelial activation by danger associated molecular patterns (DAMPs), such as low glucose and oxygen, and aggregated proteins, like Aβ and hyperphosphorylated tau (Canepa and Fossati, 2020; Freitas-Andrade et al., 2020; Sarvari et al., 2020). An increase in BBB permeability allows peripheral substances to enter the CNS, leading to neuroinflammation and oxidative stress (Pun et al., 2009; Turner and Sharp, 2016; Yang et al., 2019). Increased oxidative stress in ECs exacerbates mitochondrial dysfunction, leading to apoptosis (Lochhead et al., 2010; Fossati et al., 2012b,^2016^; Solesio et al., 2018; Parodi-Rullán et al., 2019). During both ischemic injury and AD, ECs have been observed to decrease NO production, causing dysregulation of CBF (Morikawa et al., 1994; Biernaskie et al., 2001; Girouard and Iadecola, 2006; Austin et al., 2013; Parodi-Rullán et al., 2019; Freitas-Andrade et al., 2020). Interestingly, in endothelial nitric oxide synthase (e-NOS) knockout (KO) mice, deficiency of NO reduces the ability of ECs to neutralize ROS enhancing oxidative stress and neuroinflammation, likely exacerbating AD pathology (Austin et al., 2013). Accordingly, in models of AD and stroke, EC dysfunction has been shown to enhance neuroinflammation by increasing the production of ROS and enhancing BBB permeability, amongst other cellular mechanisms (Parodi-Rullán et al., 2019, 2020; Yang et al., 2019).

### Pericytes and Smooth Muscle Cells

Pericytes and SMCs surround ECs within the vascular walls, wrapping around capillaries and arterioles/arteries, respectively. Both cell types have a vital role in the regulation of CBF and BBB integrity (Sagare et al., 2013; Hall et al., 2014; Freitas-Andrade et al., 2020). These cells regulate blood flow mainly through their contraction or dilation, which control the diameter of the blood vessel (Hall et al., 2014; Freitas-Andrade et al., 2020). Recently, the role of blood vessels in the clearance of CNS interstitial fluid and proteins has been thoroughly investigated (Aldea et al., 2019; Carare et al., 2020). Intramural periarterial drainage (IPAD) has been hypothesized as a route to drain the brain interstitial fluid and soluble proteins from the CNS into the cervical lymph nodes, by traveling along the capillaries and arterioles in the opposite direction of blood flow, within the basement membrane of the brain vasculature. The contraction of SMCs provides the motive force for IPAD (Carare et al., 2008; Aldea et al., 2019). This system is dysregulated in models of CAA and aging (Ojo et al., 2021). Another brain clearance pathway, the glymphatic system, has been observed to import cerebrospinal fluid along periarterial space and export interstitial fluid along perivenous spaces, in perivascular tunnels, formed by astroglial cells. This pathway has been proposed to be important in the clearance and regulation of toxic proteins in the brain such as tau and Aβ, and to be mediated by astrocytic aquaporin-4 (AQP4) (Braun and Iliff, 2020; Nedergaard and Goldman, 2020). Interestingly, the glymphatic system is most efficient during sleep (Boespflug and Iliff, 2018; Mestre et al., 2020).

During both stroke and AD, pericytes are observed to detach from the BBB (Sagare et al., 2013; Freitas-Andrade et al., 2020). In an AD mouse model, pericyte loss was observed to exacerbate AD pathology (Sagare et al., 2013). The loss of pericytes decreases vascular stability and decreases the ability for the NVU to regulate CBF (Sagare et al., 2013; Hall et al., 2014). On the other hand, a recent study concluded that Aβ induces pericyte-mediated capillary constriction, reducing CBF (Nortley et al., 2019). Pericytes have been observed to endure damage in models of diabetes as well as obesity, which are both risk factors of AD and IS (Price et al., 2012; Shah et al., 2013a). In cats exposed to hypoxia, pericytes exhibited detachment from the microvasculature (Gonul et al., 2002). Along with dysregulation of CBF in models of AD and stroke, pericyte and SMC loss also contribute to impaired clearance in models of AD and CAA (Sagare et al., 2013; Aldea et al., 2019; Carare et al., 2020; Kim et al., 2020; Ojo et al., 2021).

### Astrocytes

Astrocytes are essential cells for the NVU and provide a physical connection between blood vessels and neurons (Iadecola and Nedergaard, 2007; Braun and Iliff, 2020). Astrocytic end-feet wrap the blood vessels, helping to stabilize EC tight junctions and regulate CBF (Iadecola and Nedergaard, 2007). Astrocytes play a key role in providing metabolic and physical support to the CNS, along with the regulation of CBF, as well as brain clearance (Freitas-Andrade et al., 2020). AQP4 is a membrane protein that functions in water exchange within the CNS. In healthy individuals, AQP4 is a membrane channel localized at the astrocytic end feet, and it is important for clearance of toxic solutes from the brain (Smith et al., 2019). It is hypothesized that AQP4 is a part of the glymphatic system, mediating fluid exchange and the drainage of proteins, as well as the elimination of liquid from the CNS (Iliff et al., 2012; Boland et al., 2018; Braun and Iliff, 2020; Mestre et al., 2020). Despite the differential contributions of the glymphatic system and the IPAD pathway to the clearance of cerebral fluids and waste material are not completely grasped, it is accepted that astrocytes play a pivotal role in the exchange of fluids, based on studies showing that, in AQP4 KO mice, cerebrospinal fluid influx as well as CNS clearance were decreased (Iliff et al., 2012; Braun and Iliff, 2020). Another essential function of astrocytes is glutamate uptake and release, which is very important for the maintenance of CNS homeostasis (Dejakaisaya et al., 2021; van Putten et al., 2021; Verkerke et al., 2021). Astrocytes also have a critical role in the brain antioxidant system maintenance and in the production of glutathione, an important modulator of oxidative stress and aging (Bains and Shaw, 1997; Venkateshappa et al., 2012; Howarth et al., 2017; Verkerke et al., 2021).

In AD, astrocytes lose their polarization, detach from the BBB, become reactive and release inflammatory cytokines (Liddelow et al., 2017; Sweeney et al., 2019b; Cortes-Canteli and Iadecola, 2020). The crosstalk between astrocytes and microglia needs further understanding, although it has been extensively shown that they modulate each other’s activation state (Joshi et al., 2019; McConnell et al., 2019; McAlpine et al., 2021). Astrocytes have also been reported to lose their expression of AQP4 early in AD (Smith et al., 2019). Differently, in stroke, AQP4 expression correlates with cerebral edema, increasing neuronal damage (Manley et al., 2000). In IS, astrogliosis is very significant due to the presence of DAMPs during the initial/acute phase, as well as the secondary/later phase of injury. A major indicator of gliosis is an increase in the expression of glial fibrillary acidic protein (GFAP) by astrocytes, observed in stroke as well as AD models (Pekny and Lane, 2007; Oeckl et al., 2019; Freitas-Andrade et al., 2020). Upon ischemic injury, neurovascular coupling is lost, together with astrocyte mediated CBF (McConnell et al., 2019; Freitas-Andrade et al., 2020). This results in a loop of metabolic stress and inflammation, worsening mitochondrial dysfunction in vascular cells, and ultimately leading to BBB breakdown (Girouard and Iadecola, 2006; Freitas-Andrade et al., 2020; Radenovic et al., 2020; Sarvari et al., 2020).

### Microglia

Although not directly attached to ECs, microglia mediate BBB integrity (Eldahshan et al., 2019; Freitas-Andrade et al., 2020). They are considered the resident immune cells of the CNS, able to phagocytize neurotoxic substances, and, more recently, they have also been reported to participate in a series of cerebral homeostatic functions, including synaptic plasticity and brain development (Badimon et al., 2020; Willis et al., 2020). There are two well-studied activation phenotypes of microglia, referred as M1 and M2, which are classified as being pro-inflammatory and anti-inflammatory, respectively. The M1 phenotype is detrimental to the BBB, causing dysregulation of the NVU, while the M2 facilitates the endocytosis and the clearance of toxic substances and dying cells, limiting the amount of oxidative stress and promoting a more suitable environment for regeneration/healing, following CNS injury (Tang and Le, 2016; Freitas-Andrade et al., 2020; Jiang et al., 2020). Recently, a protein highly expressed in microglia, triggering receptor expressed on myeloid cells-2 (TREM2), has been observed to protect from neurodegeneration in models of AD. TREM2 is hypothesized to be neuroprotective, associated with the M2 phenotype, although there are variants that increase the likelihood of developing AD (Ulland and Colonna, 2018; Gervois and Lambrichts, 2019; Zhou et al., 2020). The protective mechanisms of TREM2 have also been observed in IS (Gervois and Lambrichts, 2019).

Following IS or HS, microglia transition into a reactive state, causing the release of pro-inflammatory cytokines, exacerbating neuroinflammation and neurovascular dysfunction (Eldahshan et al., 2019). Microglia activation is also observed in models of AD and is now considered one of the major hallmarks of the disease (Villegas-Llerena et al., 2016). Inflammasome activation has been observed to occur in aging, neurodegeneration, as well as in stroke (Mohamed et al., 2015; Liddelow et al., 2017; Hu et al., 2019; Yang et al., 2019). In the brain, inflammasome activation occurs in many different cell types. Particularly, microglia, being the most sensitive cells to pathogen associated molecular patterns (PAMPs) and DAMPs, trigger the activation of caspase-1 and the release of cytokines IL-1β along with IL-18 (Hu et al., 2019). The cytokine receptors on vascular cells are then activated, triggering detrimental processes which cause NVU dysfunction and disruption of neurovascular coupling (Eldahshan et al., 2019). Among other cells, microglia have also been observed to secrete metalloproteases (MMPs), such as matrix metallopeptidase-2 (MMP-2) and matrix metallopeptidase-9 (MMP-9), which have been reported to be activated in AD and IS, degrading the basement membrane, therefore increasing BBB permeability and NVU dysfunction (Lorenzl et al., 2003; Hernandez-Guillamon et al., 2010; Yang and Rosenberg, 2015; Turner and Sharp, 2016; Montaner et al., 2019; Freitas-Andrade et al., 2020; Sarvari et al., 2020; Carcel-Marquez et al., 2021).

### Basement Membrane

The basement membrane is an essential part of the BBB and is composed of numerous proteins such as laminins, collagen, nidogen, and heparin sulfate proteoglycans (Carare et al., 2020). The different proteins that make up the basement membrane are secreted by the cell types composing the NVU such as ECs, pericytes and astrocytes. These different proteins support cell-cell interactions, and thus BBB integrity (Carare et al., 2020; Freitas-Andrade et al., 2020).

Destruction of the basement membrane is severely apparent after middle cerebral artery occlusion (MCAO) (Sarvari et al., 2020). This degeneration occurs through different mechanisms of neuroinflammation such as ROS production as well as MMP secretion (Mohamed et al., 2015; Yao, 2019; Freitas-Andrade et al., 2020). Furthermore, the secretion and activation of MMPs interferes with the composition of the BBB, causing it to become leaky, and further exacerbating already existing oxidative stress and neuroinflammation (Fujimura et al., 1999; Hernandez-Guillamon et al., 2010; Montaner et al., 2019; Kang and Yao, 2020). Basement membrane composition and structure has also been observed to change in models of CAA (Morris et al., 2014).

### Neurons

Blood vessel-coupled-neurons communicate with ECs and other cells of the NVU to modulate vascular structure, dilation or constriction, and to provide nutrients based on neuronal need (Andreone et al., 2015; Iadecola, 2017; Kaplan et al., 2020). Neurovascular coupling is essential to maintain the proper influx of nutrients and the proper efflux of toxic waste from the brain (Girouard and Iadecola, 2006; Andreone et al., 2015; Kaplan et al., 2020). Neuronal activity has been observed to participate in both angiogenesis and neurovascular coupling (Girouard and Iadecola, 2006; Huneau et al., 2015).

Upon CNS injury and cell death of NVU cells, dysregulation of neurovascular coupling occurs (Iadecola, 2017; Sweeney et al., 2019a; Freitas-Andrade et al., 2020; Kaplan et al., 2020). The absence of communication between the CNS and the systemic blood circulation ultimately leads to metabolic failure, oxidative stress, mitochondrial dysfunction and synaptic loss (Freitas-Andrade et al., 2020; Kaplan et al., 2020). It is therefore conceivable that, when cerebrovascular integrity and function are preserved, neurodegeneration is less severe and CNS homeostasis is better maintained.

## Carbonic Anhydrases

CAs are a family of zinc metalloenzymes catalyzing the reversible reaction CO_2_ + H_2_O ⇌ HCO_3_^–^ + H^+^. All four species in this chemical reaction are essential for CNS homeostasis. Therefore, it is expected that CAs influence CNS and further NVU function. The CO_2_ hydration reaction can occur spontaneously (uncatalyzed), but the reaction rate is too slow for the dynamic of living cells. Consequently, CA is ubiquitously spread in all living organisms, including humans, where it accelerates this reaction millions of times, making the interconversion of CO_2_ and HCO_3_^–^ almost instantaneous. In humans, there are 15 CA isoforms, either acatalytic (CA-VIII, CA-X, and CA-XI) with exact function presently unknown, or catalytically active. The latter are found in the subcellular locations where CO_2_/HCO_3_^–^ interconversion is required, from the site of production in the mitochondria (mitochondrial CA-VA and CA-VB), moving into the cytosol (CA-I, CA-II, CA-III, CA-VII, CA-XIII), then to the plasma membrane (CA-IV, CA-IX, CA-XII, CA-XIV) and finally extracellularly (CA-VI secreted in saliva (Supuran, 2008; Zamanova et al., 2019; Mishra et al., 2020). As genetic manipulation and selective inhibitors (Supuran et al., 1998; Ilies et al., 2003; Smaine et al., 2007; Supuran, 2008; Güzel et al., 2009; Winum et al., 2009; Alterio et al., 2012; Akocak et al., 2016; Angeli et al., 2020) have become more available, there has been an increase in studies to determine the cell-specific function of different CA isoforms (Ghandour et al., 1992; Sly and Hu, 1995; Kida et al., 2006; Pan et al., 2006, 2012; Imtaiyaz Hassan et al., 2013; Shah et al., 2013b; Akocak and Ilies, 2014; Angeli et al., 2017; Waheed and Sly, 2017; Haapasalo et al., 2020; Mishra et al., 2020). One of the most ubiquitously expressed and catalytically active isoform is CA-II, having a turnover rate for CO_2_ hydration approaching diffusion limit (K_*cat*_ = 1.4 × 10^6^ s^–1^). It is a cytosolic enzyme and has the widest distribution in the human body, being expressed in cells from virtually every tissue or organ. In the brain, it is found in large amounts in oligodendrocytes and epithelium of the choroid plexus. Subjects suffering from CA-II deficiency syndrome, a human autosomal recessive disorder, display osteopetrosis, bone fragility, renal tubular acidosis, and, importantly, cerebral calcification and cognitive defects, developmental delay and usually a short stature (Sly and Hu, 1995; Supuran, 2008; Zamanova et al., 2019; Mishra et al., 2020). This isoform has been observed to translocate to the mitochondria, upon aging and neurodegeneration in a Purkinje cell degeneration mouse model (Pollard et al., 2016). This study also showed that *C. elegans* exposed to CA-II have a shorter lifespan, suggesting that high CA-II levels are involved in cell life/cycle-limiting mechanisms. Future studies need to further elucidate why and how this occurs.

CA-I isoform was first identified in red blood cells, where is five to six times more abundant than CA-II, although it has only about 15% of the CA-II activity (Supuran, 2008; Zamanova et al., 2019; Mishra et al., 2020). CA-I is the most abundant non-hemoglobin protein in erythrocytes and, together with CA-II, contributes to equilibration of dissolved CO_2_/HCO_3_^–^ pools in blood and maintains the pH blood homeostasis, also facilitating the CO_2_ transport from brain and metabolizing tissues to lungs (Sly and Hu, 1995; Supuran, 2008; Zamanova et al., 2019; Mishra et al., 2020). CA-I is hypothesized to contribute to cerebral edema due to the observation that CA-I is increased in the brain following HS (Guo et al., 2012). One of the most important cytosolic isozymes in the brain is CA-VII, a fast isozyme (K_*cat*_ = 9.5 × 10^5^ s^–1^), found especially in the neurons of hippocampus, together with CA-II. Interestingly, CA-VII is not found in glial cells, which contain just CA-II in the cytosol. Besides CNS, CA-VII is found in skeletal muscles, stomach, duodenum, liver, colon (Sly and Hu, 1995; Supuran, 2008; Zamanova et al., 2019; Mishra et al., 2020). In the brain, Kaila’s group has shown that CA-VII acts as a molecular switch in hippocampal CA1 pyramidal neurons in the development of synchronous gamma-frequency firing in response to high-frequency stimulation. This finding makes CA-VII an important modulator of long-term potentiation, synaptic plasticity, memory, and learning processes (Ruusuvuori et al., 2004). The same group, using a novel CA-VII (*Car7*) KO mouse model, as well as a CA-II (*Car2*) KO, and a CA-II/VII double KO mouse models, has shown that in mature hippocampal pyramidal neurons CA-VII and CA-II isozymes enhance bicarbonate-driven GABAergic excitation during intense GABA_*A*_-receptor activation. The expression of these two cytosolic isozymes was detected at a very early age in the animals (10- and 20-days post-birth), pointing toward CA-VII and CA-II being key molecules in age-dependent neuronal pH regulation (Supuran, 2008; Ruusuvuori et al., 2013; Ruusuvuori and Kaila, 2014; Zamanova et al., 2019; Mishra et al., 2020).

Membrane-bound isozymes also play an important role in the brain, especially for the regulation of extracellular pH (Chesler, 2003; Shah et al., 2005). Thus, the phosphatidylinositol glycan (GPI)-anchored isozymes CA-IV (Stams et al., 1996) is a fast isozyme (K_*cat*_ = 1.1 × 10^6^ s^–1^) found on the plasma face of the cortical capillaries. It is more resistant to inhibition by halide ions than CA-II, being adapted to perform the CO_2_/HCO_3_^–^ interconversion in the extracellular space that contains a higher concentration of Cl^–^ ions than the cytosol. The isozyme is also expressed in the choriocapillaries of the eye, in skeletal and cardiac muscles, lungs, kidneys, gastrointestinal and reproductive tracts (Sly and Hu, 1995; Supuran, 2008; Zamanova et al., 2019; Mishra et al., 2020). Another isoform localized within the plasma membrane is CA-IX, a transmembrane isozyme, possessing an N-terminal proteoglycan domain, the catalytic domain, a single-pass transmembrane region, and an intracellular tail. It is a dimeric protein with a low expression pattern in most organs, except for the digestive system (largest amount) and CNS, where it can be found mainly in the ventricular-lining cells and in the choroid plexus (Saarnio et al., 1998; Hilvo et al., 2008; Supuran, 2008; Alterio et al., 2009; Pastorek and Pastorekova, 2015; Zamanova et al., 2019; Mishra et al., 2020). The expression of CA-IX is up-regulated in hypoxia by the transcription factor hypoxia inducible factor-1 (HIF1α) and is associated with the Warburg effect in cancer pathology (Ivanov et al., 2001; Supuran, 2008; Pastorek and Pastorekova, 2015; Shabana and Ilies, 2019). In models of glioblastoma, CA-IX has been observed to increase cell migration, motility, and adhesion in monocytes (Huang et al., 2020). Currently, a compound partially selective to inhibit for CA-IX, referred to as SLC-0111, is in clinical trials, and it is likely it would operate as a therapy for multiple cancers, including glioblastoma (Boyd et al., 2017; Ilies and Winum, 2019; Shabana and Ilies, 2019; McDonald et al., 2020). This isoform has also been suggested to be involved in heart fibrosis in a rat cardiac ligation model (Vargas et al., 2016). Interestingly, in humans, CA-IX is expressed also within atherosclerotic plaques, where it is suggested to be a marker of necrotic tissue (Demandt et al., 2021). Recent studies provided evidence that CA-IX likely exacerbates cerebral ischemia outcomes (Mishra et al., 2020). CA-XII is another medium-fast (K_*cat*_ = 4.2 × 10^5^ s^–1^), membrane-bound isozyme, similar in general structure with CA-IX, but without the proteoglycan domain. It is also dimeric, with the two active sites oriented toward the extracellular milieu (Ivanov et al., 1998; Türeci et al., 1998; Whittington et al., 2001). It is found in many hypoxic tumors, alongside CA-IX, including gliomas, hemangioblastomas, and meningiomas. However, opposite to CA-IX, CA-XII is highly expressed in many normal tissues including breast epithelium and non-pigmented ciliary epithelial cells of the eye, in brain (small peripheral capillaries), esophagus, pancreas, colon, rectum, kidney, prostate, ovary, testis, endometrium, sweat glands. CA-XII was shown to play a key role in epithelial cell electrolyte homeostasis via activation of the ductal Cl^–^/HCO_3_^–^ exchanger AE2, and association with Na^+^/HCO_3_^–^ cotransporter kNBC1 (Proescholdt et al., 2005; Purkerson and Schwartz, 2007; Supuran, 2008; Hong et al., 2015; Waheed and Sly, 2017; Zamanova et al., 2019; Mishra et al., 2020). The membrane-bound isozyme CA-XIV possesses an extracellular catalytic domain, a single transmembrane helix, a short intracellular polypeptide segment, and has a moderate catalytic activity. It is highly expressed in the kidney, retina and the heart, as well as in brain, skeletal muscles, liver, and lungs. It was reported that CA-XIV is interacting with bicarbonate transporters and is involved in acid–base balance in muscles and erythrocytes in response to chronic hypoxia, and hyperactivity of the heart (Mori et al., 1999; Supuran, 2008; Mboge et al., 2018; Zamanova et al., 2019; Mishra et al., 2020).

The mitochondrial isoforms CA-VA and CA-VB are isozymes with medium-high activity (K_*cat VA*_ = 2.9 × 10^5^ s^–1^, K_*cat VB*_ = 9.5 × 10^5^ s^–1^), important for gluconeogenesis, lipogenesis, ureagenesis, and other anabolic pathways. They supply HCO_3_^–^ to pyruvate carboxylase during gluconeogenesis and lipogenesis pathways, and to carbamoyl phosphate synthetase in ureagenesis pathway. CA-VA is found mainly in the liver, while CA-VB is found in skeletal and heart muscles, kidneys, pancreas, gastrointestinl tract, brain and spinal cord (Shah et al., 2000; Supuran, 2008; Scozzafava et al., 2013; Zamanova et al., 2019; Mishra et al., 2020). Due to their involvement in these anabolic pathways, they have been studied in models of obesity and type 2 diabetes (De Simone and Supuran, 2007; Supuran, 2012; Scozzafava et al., 2013; Salameh et al., 2019; Mishra et al., 2020). The function of CA-V and the difference between CA-VA and CA-VB isoforms has been examined by analyzing the phenotypical differences between CA-VA and CA-VB KO, as well as the double KO mouse models, indicating their role in ammonia detoxification (Shah et al., 2013b). The function of CA-V in the brain is extremely understudied, however, CA-VA has been reported to be expressed in both neurons and glial cells (Ghandour et al., 2000). Interestingly, the effect of CA-V has been also investigated in cerebral pericytes (Price et al., 2017). It has been observed that silencing of both isoforms protect against high-glucose-induced cell death and ROS production, CA-VA to a more significant degree (Price et al., 2017), confirmed also by increased oxidative stress and apoptosis in models of CA-VA over expression (Price et al., 2017).

Although extensive studies reported the multiple functions of the different CA isoforms in a variety of tissues, further investigation regrading CAs cell-specific expression/activity, especially within the CNS, and in CNS disorders, is needed. In particular, CAs impact on cerebrovascular dysregulation occurring during IS and AD must still be elucidated. It is therefore crucial to identify the cell- and isoform-specific roles, and to design isoform-selective inhibitors, which may be likely to ameliorate the cell/tissue specificity and to decrease the observed side effects of the FDA-approved pan-CAIs (Provensi et al., 2019; Zamanova et al., 2019; Mishra et al., 2020).

Below, we summarize the available literature highlighting the positive effects of CA inhibition on neurovascular dysfunction in stroke-, AD-, CAA-, and diabetes-induced cerebrovascular pathology.

### Carbonic Anhydrase Inhibitors and Cerebrovascular Pathology

CAIs have been studied for decades (Maren, 1967; Bertini and Luchinat, 1983; Silverman and Lindskog, 1988; Supuran et al., 2003; Krishnamurthy et al., 2008; Supuran, 2008; Winum et al., 2009; Alterio et al., 2012; Akocak and Ilies, 2014; McKenna and Supuran, 2014; Supuran and Winum, 2015; Ilies and Winum, 2019; Angeli et al., 2020; Supuran and Capasso, 2020). They were first developed as diuretics due to their function on the reabsorption of sodium and water within the kidney (DuBose, 1984; Purkerson and Schwartz, 2007; Supuran, 2018), and are currently used to treat glaucoma as they decrease intraocular pressure, and for the prevention of high altitude sickness (Bradwell et al., 1992; Mincione et al., 2007; Ritchie et al., 2012), as they diminish pulmonary vasoconstriction, increase CBF likely controlling cerebral oxygenation, and reduce cerebral edema (Mishra et al., 2020). However, the molecular mechanism responsible for the effects of CAIs, including some of the most used pan-CAIs such as ATZ and MTZ, are multiple, and still under investigation.

MTZ, for example, was recognized as one of a few compounds that had the ability to inhibit cytochrome c release from the mitochondria under oxidative stress (Wang et al., 2008). Over 1000 compounds of the NINDS drug library were first screened in isolated mitochondria from mouse liver and further confirmed in striatal cells in models of Huntington’s disease. MTZ, inhibiting cytochrome c release from challenged mitochondria, also resulted in the reduction of caspase-9 and caspase-3 activation (Wang et al., 2008; Fossati et al., 2016; Sekerdag et al., 2018). Following this study, FDA-approved CAIs such as MTZ, ATZ, topiramate and more recently developed non-FDA approved selective inhibitors, are starting to be applied in models of cerebrovascular pathology, ischemia, CAA and AD, and their positive effects have been attributed, at least in part, to their ability to prevent mitochondrial dysfunction in cerebrovascular cells (Wang et al., 2009; Shah et al., 2013a; Fossati et al., 2016; Solesio et al., 2018).

CA inhibition has been shown to contribute to cerebrovascular tone during transient phases of pH change, but not in steady-state conditions, in rat arteries (Rasmussen and Boedtkjer, 2018). This study used two different CAIs, ATZ and 4-aminomethylbenzenesulfonamide (AMB). It was revealed that only the pan-CAI ATZ, potent against most of intracellular CA isozymes, had an effect on intracellular acidification mechanisms. Based on the observed results, it was concluded that intracellular CAs are responsible for modifying the rate of intracellular pH and vascular tone within the arteries (Rasmussen and Boedtkjer, 2018). Other studies have observed the vasodilator abilities of ATZ, such as increased NO production leading to increased blood flow in the cortex of rats (Tuettenberg et al., 2001). It is likely that the production of NO is not the only mechanism by which ATZ mediates vasodilation (Kiss et al., 1999). Systemic administration of ATZ has also been widely used as a short test to increase CBF in human studies (Okudaira et al., 1995; Grossmann and Koeberle, 2000; Russell et al., 2008). Interestingly, its stimulatory effect on CBF is reduced in patients with AD or vascular dementia compared to healthy controls (Stoppe et al., 1995; Pavics et al., 1999).

### Carbonic Anhydrase Inhibition in Models of Ischemic Stroke

The neuroprotective effects of MTZ, in both *in vitro* and *in vivo* models of ischemic injury, displayed in Table 1, were first explored by Wang et al. (2009). Exposing primary cortical neurons to OGD and H_2_O_2_ resulted in necrosis, which was rescued by MTZ treatment. Similarly, OGD increased cytochrome c release and apoptosis inducing factor (AIF) release from the mitochondria, as well as the activity of caspase-3 (Wang et al., 2009), and MTZ attenuated these effects (Wang et al., 2009), suggesting that MTZ does not only prevent necrosis, but also mitochondria-mediated apoptosis, induced by OGD. In the same study, primary cortical neurons exposed to OGD presented inflammasome activation, measured by the activation of caspase-1 and release of IL-1β. MTZ inhibited both IL-1β release as well as caspase-1 activation *in vitro* (Wang et al., 2009). This finding supports the hypothesis that CAs may mediate both apoptotic and inflammatory mechanisms. In a mouse model of MCAO, MTZ-treated mice (20 mg/kg) had a smaller infarct size, improved neurological score, and decreased cytochrome c release and caspase-3 activation, compared to non-treated mice (Wang et al., 2009). A more recent study performed in rats determined the effectiveness of ATZ alone, as well as in conjugation with head-down tilt, a way to physically promote CBF, in a transient MCAO rat model. The results indicated that ATZ was protective following transient MCAO, significantly decreasing infarct size (Han et al., 2020). Furthermore, ATZ reduced AQP4 expression, compared to rats with no treatment, suggesting a reduction in brain edema (Han et al., 2020). In a rat model of permanent MCAO, ATZ was used in comparison with selective inhibitors for CA-VII, CA-IX, and CA-XII to determine whether these isoforms are involved in the pathology. Interestingly, the pan-CAI ATZ did not improve neurological score 24 h following occlusion, however, the CAI with selectivity for CA-VII, as well as the compound more selective for membrane isoforms CA-IX and CA-XII, improved the neurological deficits observed in the occluded untreated rats. In the group treated with the CA-IX/CA XII potent and medium selective inhibitor, 6-(benzyloxy)benzo[d]thiazole-2-sulfonamide (BBT), infarct size was significantly reduced, while no reduction was observed in the CA-VII inhibitor group. However, rats treated with 50 mg/kg of ATZ also displayed diminished infarct volume. This study suggests that CA-IX and possibly CA-XII may exacerbate neuronal loss along with neurological and vascular deficits, following ischemic insult (Di Cesare Mannelli et al., 2016). Very recently, another study evaluated different CA-selective inhibitors, in both *in vitro* and *in vivo* models of IS (Dettori et al., 2021). This study analyzed a new generation of CAIs, which are lipophilic and selective for the hypoxia-associated (CA-IX and CA-XII) and cytosolic (CA-II and CA-I) isoforms. Importantly, ATZ was used as a reference compound at a much lower dose in this study (4.4 mg/kg). *In vitro*, the lipophilic CAIs and ATZ protected against OGD-induced anoxic depolarization, providing mechanistic information. *In vivo*, the CAIs reduced infarct volume, neurological deficits, neuronal damage and microglial activation (Dettori et al., 2021). This study also measured TNF-α and Il-10 plasma levels, showing differences between the sham surgery and the occluded groups, without any affect in the treated groups, likely due to the short time window of treatment (24-h). As proposed in the discussion, it is possible that, with a longer treatment, CA inhibition may mediate inflammatory pathways (Bulli et al., 2021; Dettori et al., 2021). Indeed, CAs have been also associated with inflammatory pathologies, such as rheumatoid arthritis and cancer metastasis (Supuran, 2008; Alver et al., 2011; Liu et al., 2012). Overall, both MTZ and ATZ are protective in models of IS, however, the development of CA-IX and potentially CA-XII inhibitors may be beneficial for the treatment of both neurological and vascular disorders, following IS. The protective mechanisms observed following CA inhibition in models of IS, summarized in Table 2, seem to be superior with compounds selective for membrane-bound isoforms CA-IX and CA-XII (Bulli et al., 2021).

**TABLE 1:** CA inhibition in models of IS.

Model	Mechanism	CA Inhibitor/Isoform	References
Mouse primary cortical neurons	Inhibition of OGD induced necrosis	MTZ	Wang et al., 2009
	Inhibition of OGD induced mitochondria mediated apoptosis		
	Inhibition of OGD induced inflammasome activation		
C57BL/6J Mouse pMCAO	Reduction of infarct size	MTZ	Wang et al., 2009
	Improvement of neurological score		
	Reduction of caspase 3 activation		
	Decrease of cytochrome C release		
Wistar rat tMCAO	Decrease of infarct size	ATZ	Han et al., 2020
	Reduction of AQP4 expression		
	Reduction of brain water content and sodium accumulation		
Sprague dawley rat pMCAO	Improvement of neurological score	CA-VII inhibition, CA-IX/XII inhibition	Di Cesare Mannelli et al., 2016
	Reduction of infarct size	ATZ, CA-IX/XII inhibition	
Rat hippocampal slices	Inhibition of OGD induced anoxic depolarization	ATZ, CA-IX inhibition, CA-XII inhibition	Dettori et al., 2021
Wistar rats pMCAO	Reduction of infarct size	ATZ, CA-IX/-XII inhibition	Dettori et al., 2021
	Improvement of neurological score		
	Attenuation of microglia activation		

*MTZ, Methazolamide; ATZ, Acetazolamide; HS, hemorrhagic stroke; p/Tmcao, permanent/transient middle cerebral artery occlusion; CA, carbonic anhydrase; AQP4, Aquaporin-4; OGD, oxygen glucose deprivation.*

**TABLE 2:** CA inhibition in models of HS.

Model	Mechanism	CA Inhibitor/Isoform	References
Sprague-dawley rats intracaudate blood injection	Improvement of neurological outcome	ATZ	Guo et al., 2012
	Reduction of neuronal death		
	Exacerbation of brain water content	CA-I injection	Guo et al., 2012
	Increase neurodegeneration		
Mouse primary cortical neurons	Inhibition of blood/hemoglobin induced cell death	MTZ	Li et al., 2016
	Inhibition of blood/hemoglobin induced ROS production		
C57BL/6J Mice SAH	Reduction in caspase-3 activation and cell death in hippocampus/cortex	MTZ	Li et al., 2016
	Improvement in neurological outcome		
New Zealand White Rabbits SAH	Reduction of neurodegeneration/apoptosis in hippocampus	Topiramate	Seçkin et al., 2009

*ATZ, acetazolamide; MTZ, methazolamide; CA-I, carbonic anhydrase-1; SAH, subarachnoid hemorrhage; ROS, reactive oxygen species.*

### Carbonic Anhydrase Inhibition in Models of Hemorrhagic Stroke

The protective mechanisms of CA inhibition in models of HS are summarized in Table 2. Inhibition of CA reduced brain injury after ICH in Sprague-Dawley rats (Guo et al., 2012). This study focused on CA-I, which is highly expressed in red blood cells. Upon intracaudate injection of blood, CA-I levels were increased in the ipsilateral basal ganglia, for as long as 3 days post-injection. Moreover, following intracaudate injection of CA-I, increased brain water content, microglial activation and neuronal cell death were detected, suggesting that CA-I expression may contribute to cerebral edema and neuroinflammation. ATZ-treated rats exhibited reduced perihematomal edema, as well as sodium accumulation, together with decreased neuronal deficits (Guo et al., 2012). However, the cellular mechanisms responsible for the effects of CA-I on BBB integrity require further investigation. Lately, the scientific community is beginning to hypothesize that CA-I, in both the retina and brain, contributes to vascular permeability, suggesting that specific inhibitors of CA-I could be beneficial to minimize NVU dysfunction, and thus neuroinflammation around the barrier (Gao et al., 2007). Furtherly, in a SAH mouse model, mice treated with the pan-CAI, MTZ, had decreased caspase-3 activation and apoptosis in the hippocampus and cortex, compared to the non-treated group, ameliorating neurological deficits (Li et al., 2016). In primary cortical neurons, MTZ reduced hemoglobin and blood induced cell death along with production of ROS (Li et al., 2016). Topiramate, another CAI active on multiple CA enzymes, including CA-V, reduced SAH injury in rabbits (Seçkin et al., 2009).

### Carbonic Anhydrase Inhibition in Models of Alzheimer’s Disease and Cerebral Amyloid Angiopathy

Recently, the effects of CA inhibition on mitochondrial and neurovascular cell function have been tested in models of amyloidosis (Table 3). In AD and CAA, Aβ toxic aggregates accumulate around and within the cerebral vessel walls, as well as in parenchymal plaques in specific brain regions, such as the hippocampus and cortex (Ghiso et al., 2014; Cortes-Canteli and Iadecola, 2020). Our lab has shown that Aβ oligomers and protofibrils induce cytochrome c release from the mitochondria in multiple cell types of the NVU, including ECs and SMCs, neurons and glial cells, leading to caspase activation and cell death (Fossati et al., 2010, 2012a,b, 2016; Parodi-Rullán et al., 2020). In all of these neurovascular cell types, MTZ reduced mitochondrial mediated cell death triggered by Aβ (Fossati et al., 2010, 2016). Moreover, MTZ attenuated caspase-3 and caspase-9 activation in ECs, neuronal and glial cells *in vitro*, as well as caspase-3 activation *in vivo* (Fossati et al., 2016; Solesio et al., 2018). In an *in vivo* study, Aβ was injected into the hippocampus of wild-type mice, in the presence or absence of a previous intraperitoneal MTZ injection. Interestingly, MTZ treatment attenuated the activation of caspase-3 in microglia and increased the amount of NeuN positive neurons within the hippocampus, indicating that MTZ treatment had a protective effect against neurodegeneration following Aβ-injection (Fossati et al., 2016). To elucidate the specific mechanisms responsible for these protective effects, our group showed that Aβ-induced mitochondrial dysfunction, mitochondrial membrane depolarization, cytochrome C release, and H_2_O_2_ production were attenuated in neuronal and ECs, not only by MTZ, but also by ATZ, which was effective at lower concentrations (Solesio et al., 2018). Moreover, both ATZ and MTZ inhibited caspase-9 activation caused by Aβ in cerebrovascular EC, and the resulting apoptosis (Solesio et al., 2018). MTZ has been also observed to increase the activation of nuclear factor-related factor 2 (Nrf2), in models of high-altitude sickness and more interestingly, in human neuroblastoma cells and primary cortical neurons challenged with Aβ *in vitro* (Lu et al., 2020; Sotolongo et al., 2020). Nrf2-activation by MTZ increased the activity of antioxidant enzymes, such as superoxide dismutase-1 and heme-oxygenase-1, pointing to the potential downstream effects of MTZ (Sotolongo et al., 2020). These studies support the hypothesis that CA inhibition is protective to multiple cell types of the NVU, in models of amyloidosis, and highlight the necessity to test these and/or isoform-specific compounds in clinical trials for AD and CAA. New studies currently performed in our lab are also confirming the positive effects of CAIs on cognitive performance in mouse models of amyloidosis (Angiulli et al., 2018). Additional studies are in process to investigate chronic treatments with pan-CAIs in multiple models of CAA and AD, in parallel with the assessment of specific isoform inhibitors, to further elucidate the role of different CA isoforms in AD and CAA pathology.

**TABLE 3:** CA inhibition in models of AD and CAA.

Model	Mechanism	CA Inhibitor/Isoform	References
hCMEC/D3	Inhibition of Aβ induced DNA fragmentation	MTZ, ATZ	Fossati et al., 2010; Solesio et al., 2018
	Inhibition Aβ induced of cytochrome c release		
	Inhibition of Aβ induced H_2_O_2_ production		
	Inhibition of Aβ induced caspase-9 activity		
Human primary brain SMC	Inhibition of Aβ induced DNA fragmentation	MTZ	Fossati et al., 2010
	Inhibition Aβ induced of cytochrome c release		
Normal Human Astrocytes	Inhibition of Aβ induced DNA fragmentation	MTZ	Fossati et al., 2016
Human Glioma M059K	Inhibition of Aβ induced DNA fragmentation	MTZ	Fossati et al., 2016
	Inhibition Aβ induced of cytochrome c release		
	Inhibition of Aβ induced H_2_O_2_ production		
	Inhibition of Aβ induced caspase-9 activity		
Human Neuroblastoma (SHSY5Y)	Inhibition of Aβ induced DNA fragmentation	MTZ, ATZ	Fossati et al., 2016; Solesio et al., 2018
	Inhibition Aβ induced of cytochrome c release		
	Inhibition of Aβ induced H_2_O_2_ production		
	Inhibition of Aβ induced caspase-9 activity		
	Activation of Nrf2	MTZ	Sotolongo et al., 2020
Rat primary cortical neurons	Activation of Nrf2	MTZ	Sotolongo et al., 2020
C57BL/6 Mice Aβ hippocampal injection	Inhibition of caspase-3 activation	MTZ	Fossati et al., 2016
	Increase of NeuN expression in hippocampus		
	Reduction of caspase-3 activation in reactive microglia		

*hCMEC/D3, human cerebral microvasculature endothelial cells; Aβ, amyloid-beta; MTZ, methazolamide; ATZ, acetazolamide; SMC, smooth muscle cell; SHSY5Y, human neuroblastoma cells; MO59K, glioblastoma cell line; Nrf2, nuclear factor erythroid 2-related factor.*

Interestingly, although CAIs are proving to be beneficial in preventing CAA and AD pathology, CA activators have also showed beneficial effects for memory retention in acute models (Ilies et al., 2002; Canto de Souza et al., 2017; Sanku et al., 2018; Provensi et al., 2019; Blandina et al., 2020; Schmidt et al., 2020). These findings point to a relevant role of CA enzymes in the modulation of multiple pathways in contextual fear memory extinction, neurodegenerative and neurovascular pathology. They also highlight the need for a careful and specific modulation of CA enzyme’s activity, and for a further understanding of the effects of each isoform in different brain areas, cell types, and pathological conditions.

### Carbonic Anhydrase Inhibition in Models of Diabetes-Induced Cerebrovascular Pathology

Some studies have also explored the specific inhibition of the mitochondrial isoforms CA-VA and CA-VB, which recently gained interest for their role in the prevention of cerebrovascular pathology, in different models of diabetes (Price et al., 2012, 2017; Shah et al., 2013a), which contributes to AD and stroke pathogenesis (Kuźma et al., 2018; Cortes-Canteli and Iadecola, 2020; Freitas-Andrade et al., 2020; Zlokovic et al., 2020). In a streptozotocin induced diabetic CD-1 mouse model it was reported that topiramate treatment for 3 weeks increased the levels of glutathione and reduced oxidative stress in the brain. It was also observed that diabetes-induced loss of cerebral pericytes was attenuated by topiramate (Price et al., 2012). The same group confirmed these mechanisms in the brains of CA-V double KO mice, where increased levels of glutathione and decreased oxidative stress was observed compared to wild-type (Price et al., 2012). Hyperglycemia-treated cerebrovascular pericytes increased ROS production, as well as the rate of mitochondrial respiration. The inhibition of CA (particularly CA-V), with topiramate, reduced ROS production (Shah et al., 2013a). Although topiramate has a high inhibitory activity on the CA-V isoforms, it also has affinity for the other isoforms, as well as multiple off-target effects in the brain, as shown by studies that analyzed topiramate’s functions on GABA and glutamate receptors (Johnson, 2005; Mao et al., 2015). Ideally, more specific inhibitors should be developed to further investigate the functions of CA-VA/B. The same group used a plasmid to overexpress CA-VA in cerebral pericytes exposed to high-glucose to confirm the hypothesized molecular mechanisms involving CA-VA, such as ROS production and cell death, observing that topiramate was protective against these mechanisms (Patrick et al., 2015). As a proof of concept, the same group genetically knocked down CA-VA and CA-VB in cerebral pericytes (Price et al., 2017). CA-VA and CA-VB knockdown improved cell viability, compared to the control, when exposed to hyperglycemia, although the specific knockdown of CA-VA was even more effective than CA-VB knockdown in decreasing ROS production and apoptosis, in a hyperglycemic environment (Price et al., 2017). These studies reveal potential differences between CA-VA and CA-VB function in brain pericytes, which need further investigation. In a streptozotocin induced diabetic mouse model, cerebrovascular pathology was also characterized using electron microscopy. The breakdown of the BBB and its dysfunction was attenuated by topiramate treatment *in vivo* (Salameh et al., 2016). More recently, a study using a high-fat-induced diabetes mouse model, focused on the disruption of the hippocampal BBB. BBB tight junction proteins, such as ZO-1 and claudin-12, were reduced with a high-fat diet, while topiramate treatment increased their expression, along with the attenuation of oxidative stress (Salameh et al., 2019).

## Conclusion

**FIGURE 2:**
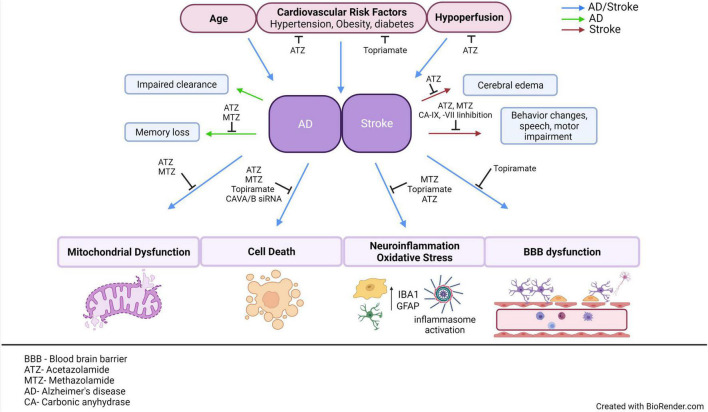
AD and stroke share common risk factors and pathological mechanisms. This figure aims to emphasize the multiple CAIs, as well as point out specific CA isoforms that have been observed to modulate cerebrovascular pathology in models of AD or stroke. Age, cardiovascular risk factors and hypoperfusion are all common risk factors between AD and stroke. Both AD and stroke exhibit NVU dysfunction, accompanied by many common molecular mechanisms, such as mitochondrial dysfunction, vascular, and neuronal cell death, neuroinflammation, and BBB dysfunction. If these mechanisms are causes or effects of neurovascular dysfunction is still a hotly debated issue. Despite the differences in the advanced pathological manifestations of the two diseases, stroke does increase the risk of dementia, suggesting that targeting their common risk factors and molecular mechanisms could ultimately mitigate the development of cerebrovascular pathology in both disorders, protecting brain health and CNS homeostasis. ATZ, Acetazolamide; MTZ, methazolamide; BBB, blood brain barrier; NVU, neurovascular unit; CNS, central nervous system; CA, carbonic anhydrase; AD, Alzheimer’s disease; CAIs, carbonic anhydrase inhibitors. This figure was created with BioRender.com.

The NVU is an important functional structure of the CNS, and its failure participates in the development of AD, vascular dementias, and exacerbates stroke outcomes. Multiple pathological and cellular mechanisms leading to NVU pathology need clarification, and new therapeutic strategies should be further investigated and developed. As scientific techniques improve, the ability to understand the functions of this unit increases. Recent studies support that CA inhibition is protective to the NVU, and the role of these enzymes should be further investigated. Table 4 emphasizes the CA isoforms that have been discussed throughout the review, their cellular localizations, their association with specific neurological disorders, and their expression in neurovascular cells. Clarifying how the cells of the NVU interact with each other, as well as the roles of CAs within each cell type, is critical for targeting cerebrovascular pathology in IS, AD, as well as other neurogenerative diseases. The pan-CAIs MTZ and ATZ, as well as topiramate, have shown protective effects in models of stroke, cerebrovascular pathology, type II diabetes, and AD, summarized in Figure 2. Although these compounds are FDA-approved, facilitating translation to clinical trials, these CAIs are not specific drugs. Isoform-specific CAIs are of increasing interest, as 15 isoforms with different functions and localizations have been identified in humans. Further preclinical and clinical studies to assess the efficacy of CA inhibition in AD as well as IS are essential. To confirm the role of specific CA isoforms as pharmacological targets, genetic studies, such as isoform KO and knock in in different NVU cell types, would be beneficial. Overall, the studies discussed above provide evidence for CAs as important potential mediators and targets in neurovascular pathology for AD, stroke and related cerebrovascular disorders.

**TABLE 4:** CA isoforms in CNS and cerebrovascular pathology.

CA isoform	Cellular localization	Cell type/Brain area	Cellular function	Neurological disorder	References
CA-I	Cytosol	Red blood cells	-pH homeostasis in the blood -Edema/sodium accumulation in the brain	IH	Guo et al., 2012
CA-II	Cytosol	-Epithelium of choroid plexus -Glial Cells -Neurons	-Intracellular ion homeostasis -Cell-life/life cycle	Loss of expression associated with cognitive abnormalities	Sly and Hu, 1995; Mishra et al., 2020
CA-III	Cytosol	MCA	N/A	N/A	Rasmussen and Boedtkjer, 2018
CA-IV	Plasma membrane	-Cortical capillaries -MCA	Extracellular pH	N/A	Chesler, 2003; Shah et al., 2005; Rasmussen and Boedtkjer, 2018
CA-VA	Mitochondria	-Cerebrovascular pericytes -Neurons -Glial cells	-High-glucose induced Apoptosis -High-glucose induced ROS production -Cell viability -Biogenesis reactions	Type-2 Diabetes induced cerebrovascular pathology	Ghandour et al., 2000; Patrick et al., 2015; Price et al., 2017
CA-VB	Mitochondria	-Cerebrovascular pericytes -MCA -CNS cells	Cell Viability	Type-2 Diabetes induced cerebrovascular pathology	Price et al., 2017; Rasmussen and Boedtkjer, 2018; Mishra et al., 2020
CA-VII	Cytosol	Hippocampal neurons	-Gamma-frequency firing -Long-term potentiation	Epilepsy	Ruusuvuori et al., 2004, 2013
CA-IX	Plasma membrane	-Glioblastoma -Choroid plexus -Ventricular linings	-Extracellular pH -Monocyte adhesion/cell migration -Tumor progression	Glioblastoma-IS	Di Cesare Mannelli et al., 2016; Huang et al., 2020; Mishra et al., 2020
CA-XII	Plasma membrane	-Glioblastoma -Peripheral capillaries -MCA	Extracellular pH	Glioblastoma	Proescholdt et al., 2005; Rasmussen and Boedtkjer, 2018

*CA, carbonic anhydrase; MCA, middle cerebral artery; IH, intracerebral hemorrhage; IS, ischemic stroke.*

## Author Contributions

NL and SF designed and conceptualized the review. NL wrote the review draft and did the literature search. SF critically revised, edited the manuscript, provided relevant insights, additional literature search, and acquired funding. EC and MI revised the manuscript and provided additional literature search. All authors contributed to the article and approved the submitted version.

## Conflict of Interest

The authors declare that the research was conducted in the absence of any commercial or financial relationships that could be construed as a potential conflict of interest.

## Publisher’s Note

All claims expressed in this article are solely those of the authors and do not necessarily represent those of their affiliated organizations, or those of the publisher, the editors and the reviewers. Any product that may be evaluated in this article, or claim that may be made by its manufacturer, is not guaranteed or endorsed by the publisher.
